# Women Suffered More Than Men Both During and After the COVID‐19 Pandemic—A Cross‐Sectional Study Among 29,079 Patients With Type 2 Diabetes

**DOI:** 10.1002/edm2.70004

**Published:** 2024-09-30

**Authors:** Grethe Åstrøm Ueland, Tony Ernes, Tone Vonheim Madsen, Sverre Sandberg, Bjørn Olav Åsvold, Karianne Fjeld Løvaas, John Graham Cooper

**Affiliations:** ^1^ Norwegian Quality Improvement of Laboratory Examinations (Noklus) Haraldsplass Deaconess Hospital Bergen Norway; ^2^ Department of Medicine Haukeland University Hospital Bergen Norway; ^3^ Department of Medical Biochemistry and Pharmacology Haukeland University Hospital Bergen Norway; ^4^ Department of Global Public Health and Primary Care, Faculty of Medicine University of Bergen Bergen Norway; ^5^ HUNT Center for Molecular and Clinical Epidemiology, Department of Public Health and Nursing, NTNU Norwegian University of Science and Technology Trondheim Norway; ^6^ Department of Endocrinology, Clinic of Medicine, St. Olavs Hospital Trondheim University Hospital Trondheim Norway

## Abstract

**Objective:**

To investigate the gender differences and the disparities between infected and noninfected patients with type 2 diabetes (T2D) regarding patient‐reported experiences during the COVID‐19 pandemic in Norway.

**Method:**

Register study using questionnaires sent electronically to patients with T2D, June 2022. The questionnaire included 82 questions covering COVID‐19 disease, symptoms, medications, comorbidities, hospital care, possibility of working from home and information received from health authorities. Clinical and demographic data were collected from the Norwegian diabetes registry for adults.

**Results:**

A total of 29,079 T2D patients participated, of whom 38.1% were women. Patients infected with COVID‐19 were younger, had shorter diabetes duration and less comorbidities than noninfected (*p* < 0.01). Women reported significantly more anxiety, depression and fear of not getting their diabetes medication than men did. Most patients were vaccinated against COVID‐19 (98.3%), whereas approximately 60% had received seasonal flu vaccine, and only 27.2% the pneumococcal vaccine. Women described more vaccine adverse effects and long Covid symptoms. Overall, 14% experienced vaccine complications and 27.3% of infected individuals reported long Covid symptoms. 2.4% of the infected patients needed hospital admission. Patients were satisfied with the follow‐up of their diabetes, and with information from the government during the pandemic.

**Conclusion:**

Female patients were more likely to experience a prolonged Covid course, and higher degree of adverse effects from the COVID‐19 vaccine than male patients. Also, long Covid symptoms were significantly more often reported among female patients, while men were more prone to be hospitalised when infected. Hospitalised patients, both men and women, had significantly higher HbA1C than those who were not hospitalised. T2D patients had a surprisingly low pneumococcal vaccination coverage, despite recommendations in national guidelines.

## Graphical abstract

Female patients with T2D were prone to a longer COVID‐19 disease course, and a higher degree of adverse effects from the vaccine, than male patients. Women also seemed to have been more psychologically affected by the pandemic, as they reported more anxiety, depression, inactivity, and weight gain.

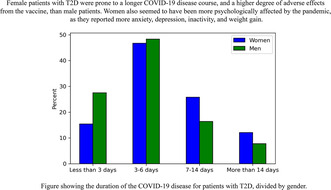

## 1 Introduction

In type 2 diabetes (T2D), elevated blood glucose levels are assumed to make individuals more susceptible to infections due to an altered inflammatory response, compounded by inflammatory mediators released by adipocytes and macrophages [1]. During infections, the patients are at risk of hypoglycaemia, hyperglycaemia and the life‐threatening condition hyperosmolar hyperglycaemia, making prompt treatment with intravenous insulin and fluids necessary. Thus, diabetes mellitus is associated with an increased risk of hospitalisation during infections [2, 3, 4, 5].

During the COVID‐19 pandemic, diabetes mellitus was early identified as one of the chronic conditions carrying increased risk of hospitalisation for COVID‐19 infections, and increased COVID‐19‐related complications and mortality [6, 7, 8]. In Norway, people with diabetes mellitus were yet not given high priority during the rollout of the COVID‐19 vaccination program. Diabetes as an underlying disease only qualified for the fifth of nine priority groups in the vaccine queue, and then only for patients above the age of 55 years. In contrast, patients with diabetes mellitus are advised to get an annual flu vaccine because of the increased risk of short‐term diabetes complications and an increased risk of pneumonia [9].

Recently our understanding of sex and gender differences in T2DM has increased. Several studies have found increased prevalence of depression, anxiety and cognitive limitations among women with T2D, and also increased prevalence of coronary heart disease, stroke and overall mortality [10]. On the other hand, men are more prone to albuminuria, impaired kidney function and foot ulcers. These factors could impact the experience of illness, disease course and outcome during intercurrent disease, like COVID‐19.

In this unique nationwide population‐based cohort study, we focus on Norwegian patients with T2D enduring the COVID‐19 pandemic. The main purpose was to study differences between infected and noninfected T2D patients regarding self‐reported severity of the disease, acute and long‐term complications of the infection, as well as the need for hospital care. Possible risk factors for severe disease and hospitalisation were also investigated, as well as vaccine status and adverse effects. The pandemic's impact on social health and the usefulness of the information given by the government were also assessed. Second, the role of gender was evaluated.

## 2 Materials and Methods

### 2.1 Participants/Data Collection

This is a register‐based study using questionnaires sent electronically to 61,708 adult patients with T2D (June 2022) included in the Norwegian diabetes registry of adults (NDR‐A). A reminder was sent after 14 days. In total, 29,079 (47.1%) answered the questionnaires. The NDR‐A contains information about the date of diagnosis, glycaemic and other risk factor control, established complications, comorbidities, medications and heredity of the patients.

The questionnaire was composed of 82 questions covering eventual COVID‐19 disease, confirmation of diagnosis with PCR or antibody testing, symptoms, medications, comorbidities, hospital care, possibility to work from home and information received from health authorities. It was created by employees in NDV‐A, with long experience in the diabetes field. Expertise from Covid researchers was consulted when needed. The questionnaire was not validated.

Long Covid was defined by symptoms persisting more than 12 weeks after the Covid infection [11].

### 2.2 Statistics

Descriptive statistics were used to quantify patient characteristics. Continuous variables were reported as mean (SD). Categorical variables were reported as number (*n*) and percentage Logistic regression analysis for binary categorical outcome variable was performed to establish the association with the demographic indicators, where the outcome variable was computed as a binary variable. A 95% confidence interval was used. Independent *t*‐test, Mann–Whitney *U* test, chi‐square and Fisher's two‐tailed exact tests were used as appropriate. *p*‐values < 0.05 were considered significant.

### 2.3 Ethics Statement

NDR‐A is an ‘opt‐out’ registry, meaning that the registry has legal permission to collect data from patients diagnosed with diabetes without the need for written consent from the patients. Patients are informed annually that they are registered in NDR‐A, and that they are allowed to opt out of the registry if so desired. The NDR‐A has ethical approval to collect patient‐reported outcome measurements (PROMs) in addition to demographic and clinical data from people with diabetes enrolled in the register. The present study has received approval from the Regional Committee for Medical and Health Research Ethics (REK Vest; Ref. No. 171685) to extract data from the register for analyses.

## 3 Results

A total of 29,079 (47.1%) individuals answered the questionnaire. The cohort consisted of 38.1% women, the median age of the population was 64 years (range 18–96). Baseline characteristics of participants are presented in Table 1. Mean HbA1C was slightly higher in men than women (56.1 [SD 12.7] versus 54.1 [SD 12.5] mmol/mol). Only 8.6% of the patients were treated with diet and exercise alone, while 30.2% were on insulin therapy alone or in combination with other antidiabetic treatments. Male participants were slightly older than women (65 versus 63 years), used more antihypertensive medications (69.1% versus 63.2%) and statins (75.0% versus 68.4%) and had more coronary heart disease (24.6% versus 9.3%) and stroke (5.1% versus 3.4%) in their medical history. The nonresponders of the study (*n* = 32,629) were older (71 versus 64 years, *p* < 0.01), and had a slightly longer diabetes duration (13.5 versus 12.5 years, *p* < 0.01) than the responders. Also, there was a higher proportion of women among nonresponders (44.4% versus 38.1%, *p* < 0.01).

**TABLE 1:** Patient characteristics.

Characteristics	T2D	Women	Men	*p*
Gender, *n* (%)	29,079	11,077 (38.1)	18,002 (61.9)	< 0.001
Age median (range)	64 (18–96)	63 (19–96)	65 (18–94)	< 0.001
Diabetes duration median (range)	12.5 (0.5–77.5)	12.5 (0.5–77.5)	12.5 (0.5–62.5)	0.5917
Comorbidities, *n* (%)				
Asthma	3598 (13.8)	1813 (18.3)	1785 (11.1)	< 0.001
Chronic obstructive pulmonary disease	1562 (6.0)	539 (5.4)	1023 (6.3)	0.003
Coronary heart disease	5454 (18.8)	1029 (9.3)	4425 (24.6)	< 0.001
Stroke	1300 (4.5)	378 (3.4)	922 (5.1)	< 0.001
Rheumatic disease	4416 (16.9)	2511 (25.3)	1905 (11.8)	< 0.001
Medications, *n* (%)				
Antihypertensive	19,358 (66.6)	6967 (63.2)	12,391 (69.1)	< 0.001
Cholesterol‐lowering drugs	20,992 (72.5)	7549 (68.4)	13,443 (75.0)	< 0.001
Prednisolone (continuous use)	815 (3.1)	325 (3.3)	490 (3.0)	0.272
Diabetic medications, *n* (%)				
Diet and exercise	2478 (8.6)	1122 (10.3)	1356 (7.6)	< 0.001
Blood sugar–lowering drugs other than insulin	16,218 (56.4)	6179 (56.6)	10,039 (56.3)	0.692
Only insulin treatment	1838 (6.4)	711 (6.5)	1127 (6.3)	0.533
Insulin and other blood sugar–lowering drugs	6840 (23.8)	2454 (22.5)	4386 (24.6)	< 0.001
Unknown	1368 (4.8)	456 (4.2)	912 (5.1)	< 0.001
Insulin regimen, *n* (% of those treated with insulin)^a^				
Insulin pen	8068 (98.6)	2897 (98.5)	5171 (98.7)	0.552
Insulin pump	114 (1.4)	44 (1.5)	70 (1.3)	0.552
Social implications of the pandemic				
Feeling anxiety during the pandemic, *n* (%)	5385 (20.6)	2635 (26.6)	2750 (17.0)	< 0.001
Feeling depressed during the pandemic, *n* (%)	8449 (32.4)	4019 (40.6)	4430 (27.4)	< 0.001
Anxiety for not getting medication during the pandemic, *n* (%)	2435 (9.4)	1243 (12.6)	1192 (7.4)	< 0.001
Wish to substitute some diabetes controls with digital follow‐up in the future, *n* (%)	5463 (25.9)	1854 (23.2)	3609 (27.7)	< 0.001
Performed more physical activity during the pandemic, *n* (%)	2583 (9.9)	1048 (10.6)	1535 (9.5)	0.005
Performed less physical activity during the pandemic, *n* (%)	6318 (24.2)	2803 (28.3)	3515 (21.8)	< 0.001
Gained weight during the pandemic, *n* (%)	3185 (12.2)	1552 (15.6)	1633 (10.1)	< 0.001

^a^ Only patients that had answered yes on the variable «only insulin treatment», or «Insulin and other blood sugar–lowering drugs» were able to answer if they used insulin pen or insulin pump.

### 3.1 COVID‐19 Infection

At the time when the questionnaire was distributed, COVID‐19 infection was reported by 8463 (29.1%) of the entire T2D cohort, which means 48.1% of the 17,594 patients who answered that they had been tested for COVID‐19. There were more women among infected patients (40.7% versus 36.4%, *p* < 0.01), and they were slightly younger than not infected ones (61 versus 64 years, *p* < 0.01). A significantly higher proportion of unvaccinated individuals were infected (60.4% versus 47.9%, *p* < 0.01). The infected T2D patients had a shorter median diabetes duration (10.5 versus 12.5 years, *p* < 0.01). We found less comorbidities among the infected patients, compared with the noninfected patients (*p* < 0.01) (Table 2), also when adjusted for age and diabetes duration. Mean HbA1c was similar in infected and noninfected individuals (55.2 versus 55.4 mmol/mol, *p* = 0.56).

**TABLE 2:** Showing characteristics between patients infected and not infected with COVID‐19.

Characteristics	COVID+	COVID−	*p*
T2D, *n* (%)	8463 (48.1)	9141 (51.9)	
Women, *n* (%)	3442 (40.7)	3323 (36.4)	< 0.0001
Age median (range)	61.0 (18.0–79.0)	64.0 (20.0–79.0)	< 0.0001
Diabetes duration median (range)	10.5 (0.5–77.5)	12.5 (0.5–62.5)	< 0.0001
Comorbidities, *n* (%)			
Asthma	1194 (14.2)	1380 (15.2)	0.062
Chronic obstructive pulmonary disease	405 (4.8)	668 (7.4)	< 0.0001
Coronary heart disease	1406 (16.6)	1817 (19.9)	< 0.0001
Stroke	310 (3.7)	467 (5.1)	< 0.0001
Rheumatic disease	1413 (16.8)	1609 (17.7)	0.121
Medication, *n* (%)			
Antihypertensive	5276 (62.5)	6089 (66.8)	< 0.0001
Cholesterol‐lowering drugs	5958 (70.6)	6569 (72.0)	0.0377
Prednisolone (continuous use)	263 (2.1)	316 (3.5)	0.188
Diabetic medications, *n* (%)			
Diet and exercise	740 (8.8)	733 (8.1)	0.087
Blood sugar–lowering drugs other than insulin	4936 (58.9)	5040 (55.8)	< 0.0001
Only insulin treatment	447 (5.3)	642 (7.1)	< 0.0001
Insulin and other blood sugar–lowering drugs	1895 (22.6)	2208 (24.4)	0.004
Unknown	363 (4.3)	417 (4.6)	0.369
Insulin pen^a^	2188 (98.4)	2656 (98.6)	0.560
Insulin pump^a^	35 (1.6)	37 (1.4)	0.560
BMI mean (SD)	29.8 (5.3)	29.6 (5.4)	0.0314
Median (min–max)	29.1 (15.0–67.1)	28.8 (15.6–72.8)	
Daily smoking	1208 (14.4)	1806 (20.0)	< 0.0001

^a^ Only patients that had answered yes on the variable «only insulin treatment», or «Insulin and other blood sugar–lowering drugs» were able to answer if they used insulin pen or insulin pump.

**FIGURE 1:**
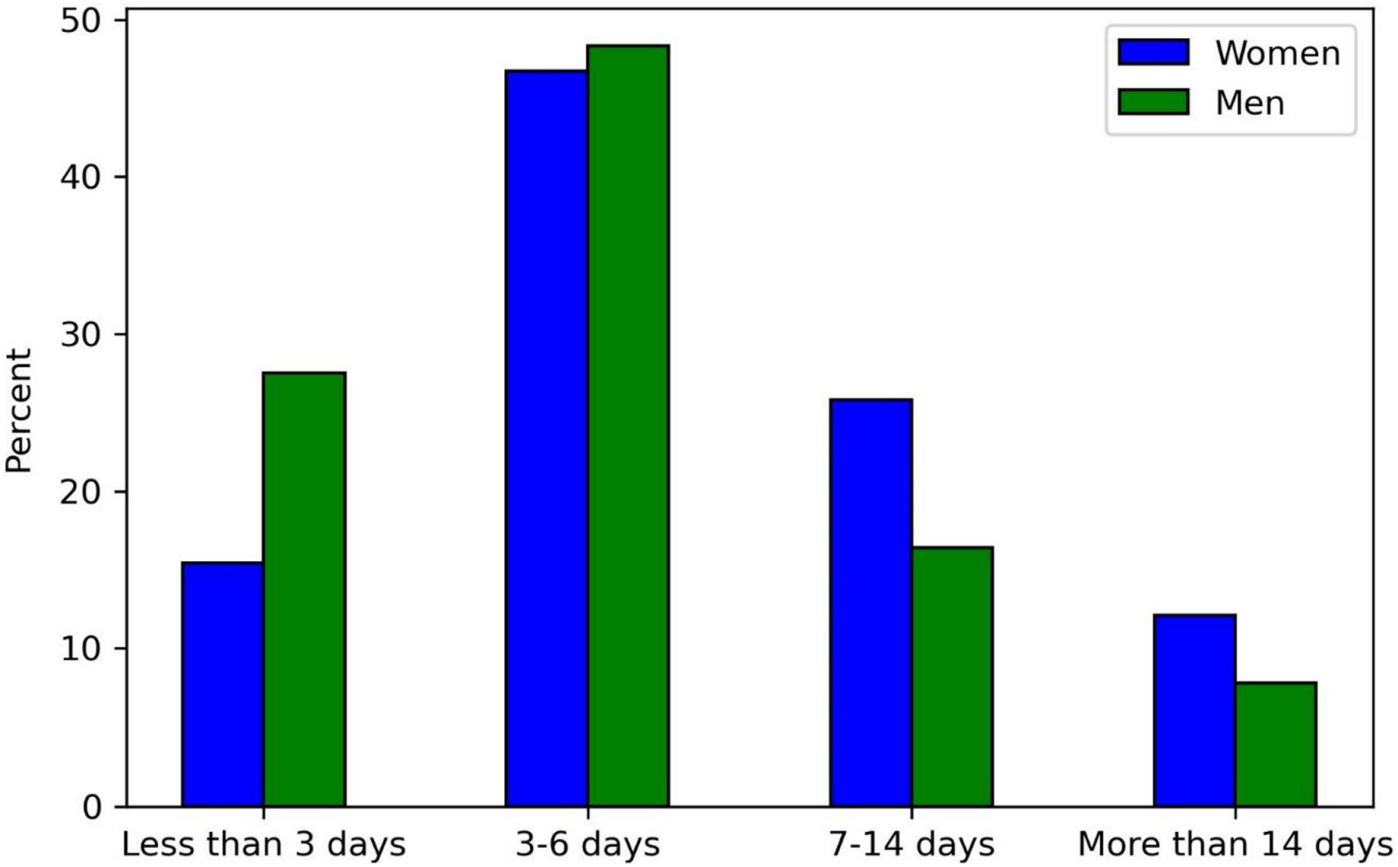
Barchart showing the duration of the COVID‐19 disease for patients with T2D, divided by gender.

Most T2D patients were sick with COVID‐19 for less than 3 days (22%) or 3–6 days (48%) (Figure 1), while 739 (10%) patients reported acute disease durations longer than 14 days. As shown in Figure 1, women were sicker for a longer period than men.

A total of 2288 (27.3%) T2D patients reported long Covid symptoms. The most frequently reported long Covid symptoms in the T2D group were fatigue, reduced workability, heavy breathing, pain, loss of sense of smell and/or taste and weight loss (74%, 54%, 48%, 39%, 28% and 13% respectively). Women reported significantly more long Covid symptoms than men (OR 1.50 [1.36, 1.65], *p* > 0.01), also when adjusted for age and diabetes duration (OR 1.46, [1.32–1.61], *p* < 0.01). Fatigue and pain were prominent symptoms.

The majority of the T2D cohort had been vaccinated against COVID‐19 (*n* = 25,718, 98.3%), 17,705 (67.7%) had also been vaccinated against seasonal flu and 7106 (27.2%) had received the pneumococcal vaccine recommended for diabetes patients.

### 3.2 Hospital Setting

Among the infected T2D patients, 198 (2.4%) were hospitalised. Of the hospitalised patients, there were fewer women (OR 0.63 [0.46, 0.84], *p* < 0.01), they were older, and had slightly longer diabetes duration (*p* < 0.01) (Table 3). HbA1C was higher in hospitalised patients compared with those not hospitalised (60.9 versus 55.0 mmol/mol, *p* < 0.01), and when adjusted for age, gender and diabetes duration OR for hospitalisation with 10 mmol/mol increase in HbA1C were 1.22 (1.07–1.45, *p* = 0.0052). Hospitalised patients had more chronic obstructive pulmonary disease (COPD), coronary heart disease, stroke, rheumatic disease and continuous use of prednisolone. There was no discernible association between hospitalisation and BMI or smoking status. Of the hospitalised patients, 64/198 (32%) reported that they had needed ventilation treatment (noninvasive or invasive), that is, 0.8% of all infected patients with T2D.

**TABLE 3:** Characteristics of hospitalised contra nonhospitalised patients.

Characteristics	Hospitalisation+	Hospitalisation−	*p*
T2D	*n* = 198	*n* = 8230	
Women, *n* (%)	60 (30.3)	3370 (40.9)	0.0026
Age median (range)	63.5 (30.0–78.0)	60 (18–79)	0.004
Diabetes duration median (range)	12.5 (1.5–38.5)	10.5 (0.5–77.5)	0.0003
Comorbidities, *n* (%)	—	—	—
Asthma	37 (18.9)	1151 (14.1)	0.0560
Chronic obstructive pulmonary disease	28 (14.3)	375 (4.6)	< 0.0001
Coronary heart disease	64 (32.3)	1336 (16.3)	< 0.0001
Stroke	15 (7.6)	293 (3.6)	0.0028
Rheumatic disease	46 (23.5)	1363 (16.6)	0.0114
Medication, *n* (%)	—	—	—
Antihypertensive	134 (68.0)	5117 (62.3)	0.104
Cholesterol‐lowering drugs	148 (75.1)	5786 (70.5)	0.160
Prednisolone (continuous use)	17 (8.7)	245 (3.0)	< 0.0001
Diabetic medications, *n* (%)	—	—	—
Diet and exercise	7 (3.6)	728 (8.9)	0.0084
Blood sugar–lowering drugs other than insulin	78 (39.6)	4837 (59.4)	< 0.0001
Only insulin treatment	33 (16.8)	413 (5.1)	< 0.0001
Insulin and other blood sugar lowering drugs	71 (36.0)	1816 (22.3)	< 0.0001
Unknown	8 (4.1)	355 (4.4)	0.8408
Insulin pen^a^	97 (98.0)	2084 (98.4)	0.7189
Insulin pump^a^	2 (2.0)	33 (1.6)	0.7189
BMI mean SD	30.7 (5.7)	29.8 (5.3)	0.0134
Daily smoking	26 (13.1)	1178 (14.5)	0.5931

^a^ Only patients that had answered yes on the variable «only insulin treatment», or «Insulin and other blood sugar–lowering drugs» were able to answer if they used insulin pen or insulin pump.

### 3.3 Adverse Effects and Complications Related to COVID‐19 Vaccine

Ninety‐eight per cent of the patients were vaccinated against COVID‐19, whereof 14% (*n* = 3605) experienced vaccine complications (Table 4). Most of the patients said they had experienced mild symptoms (*n* = 2950, 86.3%), and 7.0% (*n* = 218) reported that they had been seriously ill. Vaccine adverse effects were more common in women (20.3%) than in men (10.2%) (OR 2.26, 95% CI 2.11–2.43, *p* < 0.01). Older people experienced less vaccine adverse effects, as each extra year of age reduced the risk of experiencing adverse vaccine effect by 6.1% (95% CI). HbA1c was similar in patients with and without vaccine adverse effects (55.5 versus 55.0 mmol/mol, *p* = 0.20).

**TABLE 4:** Complications from vaccine, from Covid infection or long Covid symptoms.

Complications	T2D	Women	Men	*p*
Vaccinated for Covid	25,718 (98.3)	9755 (97.9)	15,963 (98.5)	0.001
Got vaccine complications^a^	3605 (14.0)	1974 (20.3)	1631 (10.2)	< 0.001
Mild symptoms^b^	2950 (86.3)	1584 (85.3)	1366 (87.5)	0.063
Became bedridden^b^	1011 (31.2)	632 (35.9)	379 (25.5)	< 0.001
Became seriously ill^b^	218 (7.0)	121 (7.2)	97 (6.6)	0.528
Tested for covid	17,691 (67.7)	6802 (68.4)	10,889 (67.2)	0.052
Covid positive	8463 (48.1)	3442 (50.9)	5021 (46.3)	< 0.001
Covid‐related acute complications^c^	511 (6.1)	231 (6.7)	280 (5.6)	0.031
Breathing problems that required intensive care with ventilator support	64 (12.6)	28 (12.1)	36 (12.9)	0.779
Pneumonia requiring antibiotic treatment^d^	75 (14.7)	26 (11.3)	49 (17.6)	0.047
Other infections requiring antibiotic treatment^d^	62 (12.2)	22 (9.6)	40 (14.4)	0.098
Other complications^d^	337 (66.5)	154 (67.2)	183 (65.8)	0.736
Long Covid complications^e^	2288 (27.3)	1092 (32.1)	1196 (24.0)	< 0.001
Heavy breathing^f^	1071 (47.7)	480 (44.9)	591 (50.2)	0.0111
Fatigue^f^	1654 (73.7)	825 (77.0)	829 (70.7)	< 0.001
Weight loss^f^	280 (12.6)	122 (11.5)	158 (13.5)	0.149
Change of taste/smell^f^	633 (28.2)	330 (31.0)	303 (25.7)	0.006
Pain^f^	863 (38.5)	494 (46.3)	369 (31.3)	< 0.001
Reduced workability^f^	1218 (54.1)	611 (57.2)	607 (51.3)	0.005

^a^ Proportion of vaccinated patients that experienced vaccine adverse effects.

^b^ Proportion of patients that got vaccine complications, who experienced each symptom. Several alternatives possible.

^c^ Proportion of patients infected with Covid that got acute complications.

^d^ Proportion of patients with acute complications that experienced each symptom. Several alternatives possible.

^e^ Proportion of patients infected with Covid that got long Covid symptoms.

^f^ Proportion of those that experienced long Covid who developed each symptom. Several alternatives possible.

### 3.4 Social Implications of the Pandemic

From the T2D cohort aged ≤ 67 years, 4664 (41.7%) reported that they had worked from home, at least partially. A total of 22,904 (88.0%) were satisfied with the information from the government during the pandemics.

In total, 20.6% of the patients reported that they had experienced anxiety, and 32.4% reported depressive symptoms during the pandemic. Women reported significantly more anxiety (OR 1.77 [1.66, 1.87], *p* < 0.01), depression (OR 1.81 [1.72, 1.91], *p* < 0.01) and fear of not getting their medications, (OR 1.80 [1.66, 1.96], *p* < 0.01) than men during the pandemic. They also said that they had been more inactive (OR 1.42 [1.34, 1.50], *p* < 0.01) and a higher frequency of women compared to men reported that they had gained weight (OR 1.65 [1.53, 1.78], *p* < 0.01) in this period. Based on experiences from the pandemic, 25.9% of the T2D patients were positive to replace some diabetes clinical controls with a digital solution (phone, video), more men than women (27.7% versus 23.2%, *p* < 0.01).

Of the T2D patients, 76% reported to be very satisfied with the follow‐up of their diabetes from primary health care during the pandemic. There was no difference in the prevalence of long Covid, or anxiety and depression during the pandemic between vaccinated and unvaccinated individuals, but the unvaccinated group reported lower satisfaction with the government contra vaccinated individuals (52.2% versus 88.6%, *p* < 0.01). There was no significant difference in social implications between infected and uninfected individuals.

## 4 Discussion

We present a unique, large nationwide cross‐sectional study describing T2D patients' experiences of the COVID‐19 pandemic. In summary, we found that there were more women among infected patients, and they were slightly younger than those not infected. The infected patients had a shorter median diabetes duration and less comorbidities compared with the noninfected patients. Women were sick for a longer period and experienced a higher degree of vaccine adverse effects and long Covid symptoms than men. Women experienced more anxiety, depression and fear of not getting their medications, and they also reported more inactivity and weight gain during the pandemic. On the other hand, men were to a larger degree hospitalised during COVID‐19 infection, and they also had more diabetes‐related comorbidity. Hospitalised patients had slightly higher HbA1c than those not hospitalised for both genders. Despite anxiety and depressive symptoms during the pandemic, patients were satisfied both with the follow‐up of their diabetes disease, and with information from the government.

The largest gender differences were found regarding self‐reported anxiety, depression and fear of not getting required medication during the pandemic, where women had significantly higher odds of experiencing such symptoms than men. This is in line with findings from population‐based studies of individuals with diabetes in the Norwegian background population, where especially depression was prominent in women with diabetes [12]. Our findings highlight the importance of awareness and management of psychological symptoms in women with diabetes, particularly in social crises like the COVID‐19 pandemic.

The higher proportion of younger patients in the infected group may suggest that younger individuals were less fearful of COVID‐19 and, therefore, did not impose the same level of restrictions on themselves. This is in line with our earlier published data on fear of COVID‐19, which showed increasing fear with increased age in T1D patients [13]. Less asthma, coronary heart disease and stroke among the infected patients could imply that the risk groups defined by the government showed extra caution. Another explanation could be that these patients already were identified as risk groups of COVID‐19 because of their comorbidities, and therefore, were prioritised for vaccine, and because of that avoided infection or experienced a very mild disease that was not clinically detected.

As many as 27% of the patients experienced long Covid symptoms. Fatigue, heavy breathing, loss of smell and/or taste were the most prevalent symptoms, as well as reduced workability. This is much less than findings from the background Norwegian population, where long‐term complication after COVID‐19 infection were registered in 189/312 (61%) patients 6 months after COVID‐19 infection [14]. Especially home isolated younger individuals were troubled with loss of taste and smell, fatigue, dyspnoea and impaired concentration in that study. Since patients in this study were interviewed prospectively 6 months after COVID‐19 infection, the estimates are not directly comparable.

Regarding seasonal influenza vaccine, our data are similar to a report on vaccine status from the United States in 2016, where more than 60% of diabetic patients had received the vaccine in the past year [15]. Regarding the pneumococcal vaccine, we were surprised that only 28% of T2D patients had received the vaccine in our study, a much lower coverage than in the United States where 52.6% of diabetic patients had received the pneumococcal vaccine [15]. Our conclusion is that information about this vaccine recommendation is suboptimal in Norway.

Eighteen per cent of our patients reported that they had experienced adverse effects from the COVID‐19 vaccine. This is considerably less than that reported from PROMs data in Saudi Arabia, where 60% experienced side effects due to the COVID‐19 vaccines [16]. Fatigue and pain/redness at the injection site were the most reported side effects among their study participants (90% and 85% respectively). Sixty‐six per cent of the participants reported fever and 36% of them reported having chills. Headache was common among the individuals in the study (62%). However, nausea, vomiting, joint and bone pain were less commonly reported by the study participants [16]. The main difference from our data is probably that we did not ask for mild symptoms like pain and rubor at the injection site, and hence more serious adverse effects are reported in our study.

2.4% of the infected patients needed to be admitted to hospital during Covid infection, men significantly more than women. For comparison, an analogous study of Addison disease in Sweden showed that a larger proportion (8.5%) of the infected patients were hospitalised [17]. Older age, male gender and comorbidities like COPD, coronary heart disease, stroke, rheumatic disease and continuous use of prednisolone seemed to dispose for hospitalisation. Hypertension and old age have been associated with an increased risk of severe COVID‐19 in several previous studies [18], but our study only supports the old age in this setting. Among patients hospitalised for Covid, 12.6% needed mechanical noninvasive or invasive ventilation treatment. That is comparable with the Norwegian background population where 9%–12% of the hospitalised Covid patients needed such treatment [14, 19]. However, among all infected diabetes patients, only 0.8% needed ventilation treatment during their Covid infection, which is lower than in the general Norwegian population (where 1.7% needed ventilation support) [19].

Patients were generally satisfied with their diabetes follow‐up both in primary care and specialist care. Also, we found that patients with diabetes mellitus reported satisfaction with the information received from healthcare authorities and the government. This is in line with a publication from Chen et al., which scrutinised important factors influencing the satisfaction of citizens concerning their government responses to the COVID‐19 pandemic in 14 countries, including Sweden and Denmark [20]. They found that people's satisfaction was closely related to confirmed cases of infection and deaths per million, more than the measures that were implemented to achieve this. Norway have had low infection and death numbers throughout the pandemic [21].

The primary strength of this study is the large number of participants in different age groups from the whole of Norway. Previous data from the NDR‐A show quite similar frequency of risk factors and complications, which means it can be assumed that the cohort used in this study is representative for the T2D cohort of Norway. In June 2022, when the patients received the questionnaire, 1,426,267 persons were reported infected in Norway [22]. Also, the initial variants of Covid were reported to be more virulent and more aggressive than later variants.

We used a self‐reporting approach and consequently, the risk of recall bias must be considered for the presence of symptoms and severity. Yet, this study design allows for a unique insight into the self‐management of diabetes mellitus. It also has to be taken into consideration that studies have shown gender differences in health information behaviour, meaning that women have a greater tendency to be attentive to their health, which might make them more likely to identify and report symptoms, fear and findings [23]. Comparison of vaccinated and unvaccinated individuals must be interpreted with caution, as there are few unvaccinated individuals in the study. Finally, we acknowledge that fewer women than men participated in the study. This disparity may be partly attributed to the fact that NDV‐A includes a higher percentage of men than women (56% versus 44% respectively). However, there remains an unexplained lower response rate among women.

To conclude, this is a Norwegian nationwide study on self‐reported experiences of the COVID‐19 pandemic among diabetic patients. We found several gender differences. First, female patients were prone to a longer disease course, and a higher degree of adverse effects from the COVID‐19 vaccine than male patients. Women also seemed to have been more psychologically affected by the pandemic, as they reported more anxiety, depression, inactivity and weight gain. This is important knowledge if a new public health crisis appears. On the other hand, men were more susceptible to being hospitalised when infected with COVID‐19. Additionally, high HbA1c predisposed to hospitalisation in both genders. A significant proportion of patients reported long Covid symptoms, especially women suffering from fatigue and pain. Our findings support a gender‐specific approach to diabetes patients during crises like the COVID‐19 pandemic. Finally, patients were in general very satisfied with the information given, both from the general practitioner and from the government throughout the pandemic.

## Author Contributions

**Grethe Åstrøm Ueland:** conceptualization (lead), data curation (lead), formal analysis (supporting), methodology (lead) visualization (lead), writing – original draft (lead), writing – review and editing (lead). **Tony Ernes:** data curation (supporting), formal analysis (lead), methodology (supporting), writing – original draft (supporting). **Tone Vonheim Madsen:** methodology (supporting), writing – original draft (supporting), writing – review and editing (supporting). **Sverre Sandberg:** supervision (supporting), writing – review and editing (equal). **Bjørn Olav Åsvold:** supervision (equal), writing – review and editing (supporting). **Karianne Fjeld Løvaas:** conceptualization (equal), supervision (equal), writing – original draft (supporting), writing – review and editing (supporting). **John Graham Cooper:** conceptualization (equal), supervision (equal), writing – original draft (supporting), writing – review and editing (lead).

## Conflicts of Interest

The authors declare no conflicts of interest.
